# Explaining the geographic spread of emerging epidemics: a framework for comparing viral phylogenies and environmental landscape data

**DOI:** 10.1186/s12859-016-0924-x

**Published:** 2016-02-11

**Authors:** Simon Dellicour, Rebecca Rose, Oliver G. Pybus

**Affiliations:** Department of Zoology, University of Oxford, Oxford, OX1 3PS UK; Rega Institute for Medical Research, Clinical and Epidemiological Virology, Department of Microbiology and Immunology, KU Leuven, University of Leuven, Minderbroedersstaat 10, 3000 Leuven, Belgium

**Keywords:** Phylogeography, Phylodynamics, Molecular epidemiology, Rabies virus

## Abstract

**Background:**

Phylogenetic analysis is now an important tool in the study of viral outbreaks. It can reconstruct epidemic history when surveillance epidemiology data are sparse, and can indicate transmission linkages among infections that may not otherwise be evident. However, a remaining challenge is to develop an analytical framework that can test hypotheses about the effect of environmental variables on pathogen spatial spread. Recent phylogeographic approaches can reconstruct the history of virus dispersal from sampled viral genomes and infer the locations of ancestral infections. Such methods provide a unique source of spatio-temporal information, and are exploited here.

**Results:**

We present and apply a new statistical framework that combines genomic and geographic data to test the impact of environmental variables on the mode and tempo of pathogen dispersal during emerging epidemics. First, the spatial history of an emerging pathogen is estimated using standard phylogeographic methods. The inferred dispersal path for each phylogenetic lineage is then assigned a “weight” using environmental data (e.g. altitude, land cover). Next, tests measure the association between each environmental variable and lineage movement. A randomisation procedure is used to assess statistical confidence and we validate this approach using simulated data. We apply our new framework to a set of gene sequences from an epidemic of rabies virus in North American raccoons. We test the impact of six different environmental variables on this epidemic and demonstrate that elevation is associated with a slower rabies spread in a natural population.

**Conclusion:**

This study shows that it is possible to integrate genomic and environmental data in order to test hypotheses concerning the mode and tempo of virus dispersal during emerging epidemics.

**Electronic supplementary material:**

The online version of this article (doi:10.1186/s12859-016-0924-x) contains supplementary material, which is available to authorized users.

## Background

Evolutionary and phylogenetic analysis has become an important tool in the study of established and emerging viruses, including influenza e.g. [1], HIV e.g. [2], MERS e.g. [3], and most recently, Ebola [4, 5]. The application of evolutionary concepts to epidemiological surveillance and outbreak control has been transformed in recent years by the increasing availability of viral genome sequences, the growth in computer processing power, and the development of sophisticated analytical methods (e.g. [6]). Information gleaned from genetic data has the potential to identify factors that influence the spread and evolution of viral diseases. An evolutionary approach to epidemiology provides several benefits when combined with traditional methods, as it allows reconstruction of epidemic transmission history from a small number of pathogens sampled shortly after the discovery of an outbreak, the estimation of migration histories, and the inference of transmission links among cases that may not be evident using data on spatio-temporal incidence alone.

Evolutionary approaches are particularly powerful in the context of RNA viruses because these viruses are characterised by very rapid evolution [7], such that their evolutionary and ecological processes occur on the same time scale [8, 9]. As a consequence the pattern of genetic differences among viruses sampled from a population contains information about transmission history. Specifically, phylogeographic and statistical studies of virus genomes sampled through space and time can answer questions relating to the geographic dissemination of epidemics e.g. [4, 10] and numerous methods are available to infer patterns of spatial spread from genetic data, e.g. [11–15]. However, the explanatory power of viral phylogeography would be greater if it could identify and test the effects of specific aspects of the environment on spatial spread and growth. This question represents a major focus of current research in viral phylogeography.

Lemey et al. [15] developed a method based on relaxed random walk models that can reconstruct virus dispersal in continuous space using viral genomes sampled from known locations at known times. This method co-estimates both the virus phylogeny and the locations of unsampled common ancestors, thereby producing a full spatial dispersal history of the sampled infections. This history includes an estimate of the location of epidemic origin and of the velocity, direction and heterogeneity of spatial spread. The method has been used in several studies to reconstruct the spatial spread of pathogenic viruses, including crop diseases, dengue virus, West Nile virus and avian influenza viruses [16–18]. However, while this method can place phylogenies in a geographical context, it does not explicitly incorporate environmental differences across the geographic landscape within which transmission occurs. Such heterogeneity may have a significant effect on spatial dissemination, especially for outbreaks in natural animal and plant populations e.g. [19–21].

At present there is only one phylogeographic method that explicitly tests potential predictors of spatial spread. This is the “phylogeographic GLM” approach [22, 23] that estimates rates of lineage movement among a fixed number of discrete locations, and in doing so parameterises each among-location rate as a linear function of one or more predictor variables. The coefficients of the linear model are then co-estimated with the among-location rates, the phylogeny, and other parameters, using Bayesian Monte Carlo Markov Chain (MCMC) inference. However, this framework is only currently applicable to discretised spatial locations and is more suited to hypotheses concerning human mobility among population centres via transportation hubs, such as airports [23, 24] and is less suitable for hypotheses concerning the dissemination of animal and plant pathogens throughout natural landscapes. Further, it is computationally demanding because predictor variables are assessed whilst sampling phylogenies using a Bayesian MCMC algorithm. There is therefore a need for complementary approaches.

Here we present and apply a new analytical framework that aims to integrate landscape ecology with a phylogenetic approach to molecular epidemiology. This framework uses spatial information obtained from phylogeography to study the impact of environmental variables on epidemic dispersal in continuous space. Our method differs from previous approaches in that it explicitly separates the task of testing correlates of lineage dispersal from the task of inferring the history of movement from genetic data. This separation has two benefits. First, it allows large numbers of environmental variables to be assessed without the need for phylogenetic MCMC sampling. Second, it increases flexibility, because it can be applied to any dispersal history, irrespective of the specific phylogeographic method or software package that was used to infer that history. We illustrate our framework by applying it to a well-characterised viral outbreak, the spread of rabies in raccoons across the north-east of the USA over approximately thirty years (hereafter referred as the raccoon rabies epidemic). This data set has been analysed using previous phylogeographic methods [15, 25], thus enabling direct comparison with the results obtained here.

## Methods

### Overview of methodology

The structure of our framework can be summarised in the following five steps. A full description of each step is provided in the next section.

**Fig. 1:**
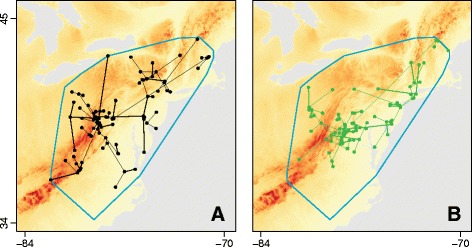
An illustration of the node position randomisation procedure used to generate null distributions of the *D* statistic. **a** The original environmental raster (representing, in this case, elevation) upon which is superimposed the movement events extracted from one spatiotemporally-referenced phylogeny. **b** The result of one randomisation of node positions. This randomisation procedure is performed within a minimum convex hull (shown in blue), which is defined by the node locations of all selected phylogenies
(ii) In a second, optional, step, the spatio-temporal data extracted in step (i) is used to calculate summary statistics of spatial spread, such as the velocity of the epidemic wavefront.
(iii) Each of the vectors obtained in step (i) is assigned a “weight” score, which is calculated using a raster that defines the spatial heterogeneity of a specified environmental variable (e.g. elevation, human population density, land cover, etc.). We have implemented three different models of spatial movement (hereafter called “path models”) to compute the “weight” allocated to each phylogeny branch for a given environmental raster. (a) The “straight-line path” model, which assumes that movement is in a straight-line between the start and end locations of each branch. (b) The “least-cost path” model, which uses a least-cost algorithm [26] to determine the route taken between the start and end points. (c) The “random walk path” model, which uses circuit theory [27] to accommodate uncertainty in the route taken. Note that for models (b) and (c), each environmental raster must be considered twice, once as a conductance factor (i.e. it facilitates movement) and once as a resistance factor (i.e. it impedes movement).
(iv) The correlation between the duration of each phylogeny branch and its “weight” (see step iii) is then estimated. Correlations are repeated for each environmental raster, and for each of the three path models described above.
(v) Finally, the statistical significance of these correlations is tested using a null model. To generate this null model, we implement a simple randomisation procedure: phylogenetic node positions are randomised within the study area, under the constraint that branch lengths, tree topology and root position are unchanged (see Fig. 1b).

(i) In the first step, the history of lineage dispersal is recovered from one or more spatial- and temporally-referenced phylogenies (i.e. trees whose branches represent time and whose tips and internal nodes all have a defined location). Such trees are generated by the continuous phylogeography method implemented in BEAST [15], but it is important to note that our method is applicable phylogenies from any source that are annotated with dates and locations in the same way (Fig. 1a). The velocity, distance and duration of spatial movement along each branch in each tree are extracted and represented by a vector.

### Description of methodology

#### Step 1: Extracting spatio-temporal information from phylogenies

The input data for our method consists of one or more spatiotemporally-referenced phylogenies (i.e. trees whose branch lengths are proportional to time and whose internal and external nodes are all annotated with a point location in two-dimensional space; Fig. 1a). If multiple phylogenies are used then each is assumed to be equally probable. At present, such trees are mostly likely to be generated using the phylogeographic models implemented in BEAST [15], but our framework can be applied to phylogenies from any source that have been annotated in the same way.

In order to extract the spatio-temporal information in the input trees, each phylogeny branch in each tree is summarised as a vector defined by its start and end location, and its start and end dates. Each branch therefore represents an independent viral lineage dispersal event [18]. Vectors for each input tree are tabulated.

#### Step 2: Estimation of dispersal and epidemiological statistics

As an optional step, our analytical framework calculates several spatio-temporal statistics from these dispersal vectors. So far, we have implemented three such statistics: the mean lineage dispersal velocity, the mean lineage diffusion coefficient, and a time-series of the maximal epidemic wavefront distance. These statistics are visually summarised as plots of (i) the kernel density of lineage dispersal velocity parameters (the mean and variation among lineages in dispersal velocity), (ii) the kernel density of lineage diffusion coefficient parameters (the mean and variation among lineages in diffusion coefficients), and (iii) the change through time in the spatial and patristic maximal wavefront distances, as measured from the location of the tree root. The spatial distance corresponds to a straight-line distance, whereas the patristic distance equals the sum of the spatial distances along each phylogeny branch between the tree root and its tips.

#### Step 3: Computation of environmental weights

In this step, each of the vectors obtained in step 1 (one per phylogeny branch) is assigned a specific “weight” based on a raster of values that represent the type or magnitude of an environmental variable (e.g. elevation, population density, type of land cover). In order to compute these “weights”, we have implemented three different “path models” which represent the path taken by a phylogeny branch as it travels between its start and end locations:

(i) *Straight-line path*: each lineage travels in a straight line between its start and end location. In this case, the branch “weight” is computed as the sum of the values of the raster cells through which the straight line passes.
(ii) * Least-cost path*: each lineage travels via the least-cost path between its start and end location [26]. With this model, the branch “weight” is computed as the sum of the transition values between adjacent cells along the least-cost path [28].
(iii) * Random walk path*: each lineage travels via a random walk between its start and end location. In this case, the “weight” is a graph-theoretic metric based on circuit theory, which takes into account multiple possible pathways connecting a given pair of locations, and the values of the raster cells through which they pass [27, 29]. Specifically, for each lineage, one node is connected to a one-ampere current source, while the other is connected to ground. The “weight” between each pair of nodes is then defined by the effective electric resistance or conductance connecting them on the grid, i.e. the environmental raster in question [27]. More details about the circuit theory model can be found in [27] and [29].

For the latter two models, an environmental variable can be treated either as a conductance or a resistance factor. For example, if an environmental raster is treated as a resistance factor then raster cells with low values will be more permeable to dispersal and those with high values will correspond to poor dispersal habitat or to movement barriers [27]. When there is no prior information about whether a given variable will facilitate or impede lineage movement, it is prudent to consider it twice, once as a potential conductance factor, and once as a potential resistance factor. By default, we use the environmental values as resistance or conductance values. Log-transformed and/or standardised values may be necessary if the current univariate approach is in future extended to a multivariate one (see Conclusion). Note, when all cells in a raster are identically-valued (i.e. there is no spatial variation in the environmental factor) then the total “weight” for any path is simply proportional to its geographic distance. A special case exists when all cells have values equal to 1, which we here define as the “null raster”.

#### Step 4: Correlation analyses

We next calculate the regression between the duration of each movement event (i.e. each phylogeny branch) and the “weight” computed for that branch. A separate regression is performed for each environmental factor of interest. The *absolute* strength of the regression can be measured using its coefficient of determination (R^2^). Specifically, we use the statistic *D* = (R^2^_env_ - R^2^_null_), where R^2^_env_ is the R^2^ obtained when branch durations are regressed against weights defined by the environmental raster, and R^2^_null_ is the R^2^ obtained when branch durations are regressed against weights defined by the null raster (i.e. when only the spatial distance of each movement event is considered). All environmental raster cell values are increased by 1 (except for cells with no data) to enable a direct comparison of R^2^_env_ and R^2^_null_. *D* is therefore a correlation measure relative to a null hypothesis and represents the degree to which the regression is strengthened when spatial variation in the environmental variable is taken into account.

#### Step 5: Significance testing using randomisation

In the final step, the statistical significance of *D* is tested. In order to calculate a null distribution for the *D* statistic, we have implemented a randomisation procedure that randomises phylogenetic node positions under the constraint that branch lengths (i.e. branch durations), the tree topology and the root position are unchanged (Fig. 1b). Furthermore, this randomisation procedure is not applied to the entire raster but to a subset of it defined by the minimum convex hull around a set of locations. The set of locations comprises the start and end positions of each branch in the spatiotemporally-referenced input phylogenies. During the randomisation, if a randomised branch position falls outside the area defined by this convex hull, the algorithm randomises its position again until it does not fall outside this area. By constraining permutation within this convex hull we are, in effect, using the data to inform the relevant study area. In contrast, the square raster inputted by the user may be of arbitrary size and orientation. Without this constraint, randomised phylogenies will fall in areas with different environmental values, potentially leading to type I errors. The result of this procedure is one *p*-value per input phylogeny, which equals the proportion of randomisation replicates that generated *D* values larger than that generated by the empirical input phylogeny. If multiple input trees are used then a distribution of *p*-values is obtained and we report the percentage of *p*-values > 0.05. It is worth noting that the statistical significance of our test is derived from a randomisation distribution, not from a distribution derived from the regression model. Hence the coefficients of determination R^2^_env_ and R^2^_null_ are treated only as data statistics, and therefore our test is not dependent on regression model assumptions (such as homoscedasticity).

### Application to rabies in North America

The raccoon rabies virus data set comprises 47 sequences with known sampling dates and locations. The sequences are ~2800 nt long and span the viral nucleoprotein *N* gene and the 5' end of the phosphoprotein *P* gene; see Biek et al. [25] for further details). We extracted the spatial-temporal information contained in 100 spatiotemporally-referenced trees sampled from the posterior distribution of trees inferred for this data set by Lemey et al. [15]. We first calculated three epidemiological statistics from these 100 input trees: mean lineage dispersal velocity, mean lineage diffusion coefficient and a plot of the maximal wavefront distance. For ease of explanation, we initially show how our analytical framework works on a single input phylogeny. Subsequently we show how the method is extended to incorporate phylogenetic uncertainty by analysing multiple input trees.

**Fig. 2:**
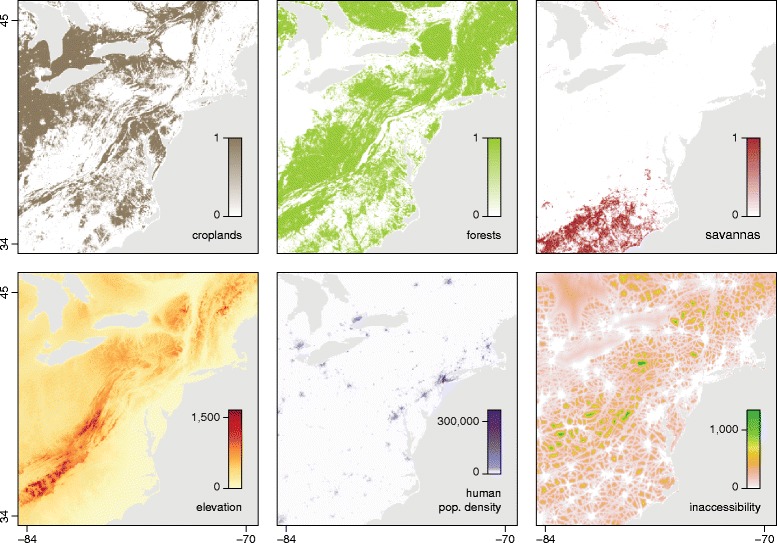
The six environmental variables that were tested in the analysis of the raccoon rabies virus data set. The region shown corresponds to the northeast of the USA, centered approximately on Harrisburg, PA. Details of the construction and source data for these rasters is provided in the main text

We investigated six environmental variables to determine if they were associated with the dispersal rate of raccoon rabies lineages. These included the three most important IGBP (International Geosphere Biosphere Programme) land cover variables for the study area (i.e. “croplands”, “forests” and “savannas”), as well as elevation, human population density and “inaccessibility” (measured as travel time to major cities of > 50,000 people [30]). The “forests” and “savannas” layers combine several IGBP land cover layers (evergreen needleleaf, evergreen broadleaf, deciduous needleleaf, deciduous broadleaf and mixed forest layers for the “forests” layer; savannas and woody savannas for the “savannas” layer). The environmental rasters are shown in Fig. 2. The sources of the original raster files are given in Table 1. The original data presented a resolution of 0.5 arcmin, corresponding to cells ~1 km square. We generated distinct land cover rasters from the original data by creating lower resolution rasters (10 arcmin) whose cell values equalled the number of occurrences of each land cover category within the 10 arcmin cells. The resolution of the three other original rasters was also decreased to 10 arcmin for tractability. For each selected raster and path model combination, we performed 100 randomisations using the randomisation procedure outlined in step 5 above.

**Table 1:** Source of data for each environmental raster

Original raster	Source	URL
Land cover	IGBP (International Geosphere Biosphere Programme)	www.igbp.net
Elevation	SRTM (Shuttle Radar Topography Mission) near-global DEMs (Digital Elevation Models)	webmap.ornl.gov
Human density	GRUMP (Global Rural–Urban Mapping Project), MAP (Malaria Atlas Project)	www.map.ox.ac.uk
Inaccessibility	Global Environment Monitoring Unit, Joint Research Centre of the European Commission	bioval.jr.ec.europea.eu

### Performance on simulated data sets

We used artificial data sets to measure the potential type I error rate of the randomisation procedure implemented to assess the significance of observed *D* values. Artificial data sets were generated using four different approaches: (i) the duration of each movement event was randomly permuted among branches, (ii) the duration of each movement event was equal to the spatial distance along the path between its start and end locations, (iii) the duration of each movement event was equal to the environmental “weight” assigned to the path between the locations, and (iv) the duration is equal to a value randomly and uniformly chosen between the values defined in cases (ii) and (iii). Case (iv) thus corresponds to case (iii) but with the addition of random noise. Simulation approaches (i) and (ii) generate data sets under the null hypothesis (i.e. branch duration is independent of the environmental raster), whereas approaches (iii) and (iv) generate data sets under the alternate hypothesis (i.e. branch duration is determined or influenced by the environmental raster).

We generated 100 artificial data sets for each of these three simulation approaches. The artificial data sets used the empirical phylogenies from the raccoon rabies data set (see above), but replaced the empirical branch durations with new durations as specified above. For approach (iii), environmental “weights” were calculated using the “elevation” raster treated as a resistance factor. This raster was chosen because elevation appears to impede the movement of raccoon rabies virus lineages (see Results). For each path model and simulation method, we report the percentage of artificial data sets for which the randomisation null hypothesis test *p*-value was < 0.05. For simulation approaches (i) and (ii), these percentages correspond to estimates of the type I error rate of the test. For approaches (iii) and (iv), these percentages indicate statistical power.

## Results and Discussion

We use the methods outlined above to analyse previously published virus gene sequences sampled from an epidemic of rabies virus in raccoons in North America [25]. Spatio-temporal phylogenies were estimated from these sequences using the continuous phylogeographic model implemented in BEAST [15]. The analyses below were performed on 100 phylogenies sampled regularly from the post burn-in posterior tree distribution generated by BEAST.

### Estimation of spatio-temporal statistics

**Fig. 3:**
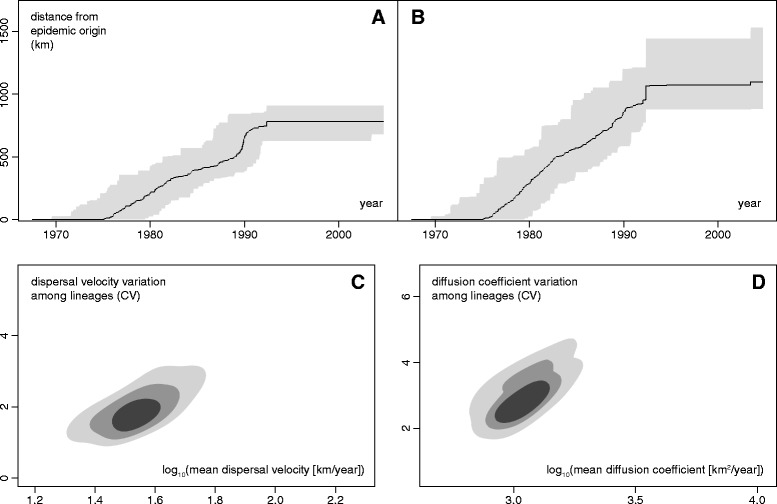
Epidemiological statistics estimated from the raccoon rabies virus data set. **a** Time-series of the spatial distance between epidemic origin and maximal epidemic wavefront, and **b** evolution of the patristic distance between epidemic origin and maximal epidemic wavefront, **c** kernel density estimates of lineage velocity parameters and **d** kernel density estimates of lineage diffusion coefficient parameters (coefficient of variation “CV” against mean values). In parts **a** and **b** the grey area corresponds to the 95 % credible region of the estimated wavefront position. In parts **c** and **d** the three contours show, in shades of decreasing darkness, the 25 %, 50 %, and 75 % highest posterior density regions via kernel density estimation

We used our framework to calculate several statistics that summarise the spatio-temporal information inherent in the rabies virus phylogenies (see Step 2 of Methods for details). These statistics include the epidemic wavefront and patristic distances through time (Figs. 3a and b), and parameters that describe dispersal velocities and diffusion coefficients (Figs. 3c and d). The epidemic wavefront plot (Fig. 3a) shows that the spatial extent of the raccoon rabies epidemic increased at a relatively constant rate until around 1990. The patristic distance (i.e. the distance summed along tree branches) at this time is greater (> 1,000 km) than the spatial distance (750–800 km). The difference between these two measures is to be expected, because the patristic distance includes movements in all directions, including those back towards the epidemic origin (Fig. 3b). Hence dispersal was diffusive in nature and did not always follow the shortest or most direct path from epidemic origin to epidemic wavefront. Kernel density graphs of the mean lineage velocity (Fig. 3c) and phylogenetic diffusion coefficient (Fig. 3d) indicate significant variation in these parameters among lineages. The estimated mean lineage velocity was ~37 km/year (Fig. 3c), similar to that reported for dengue virus in Vietnam (6–38 km/year [17]) but substantially smaller than that of the West Nile virus epidemic in North America (1,500 km/year [18]). These statistics are helpful in quantifying the spatial dynamics of an epidemic and can also be used to compare different outbreaks. Some, but not all, of them are implemented in the software package SPREAD [31]. We hope that future work will lead to the development and implementation of further summary statistics.

### Impact of environmental variables

Next, we used our framework to measure and test the correlation of various environmental variables with the movement of rabies virus lineages (see Steps 3–5 in Methods). We explored rasters that represent six different environmental variables, specifically (i) cropland land cover, (ii) forest land cover, (iii) savanna land cover, (iv) elevation, (v) human population density and (vi) inaccessibility to major cities. For illustrative purposes, we first present the results based on only one tree (from the posterior phylogeny distribution estimated by BEAST). We later show how the framework can be extended to incorporate phylogenetic uncertainty, by analysing multiple input trees.

The purpose of the analysis is to calculate a test statistic, *D,* for each environmental variable, *E*. The statistic *D* represents the degree to which the correlation between the duration and “weight” of each lineage is strengthened when spatial heterogeneity in *E* is taken into account (see Steps 3–4 in Methods for details). Formally, *D* = R^2^_env_ - R^2^_null_, where R^2^_env_ is the coefficient of determination obtained when branch durations are regressed against branch weights calculated using raster *E*, and R^2^_null_ is the coefficient of determination obtained when branch durations are regressed against branch weights defined by a null raster with no spatial variation (i.e. a raster whose cells all have value 1). If *D* ≤ 0, then the environmental variable *E* does not explain variation in branch duration better than geographic distance alone. However, if *D* is strongly positive, then the values of *E* are strongly associated with branches that move more rapidly or more slowly than average.

**Fig. 4:**
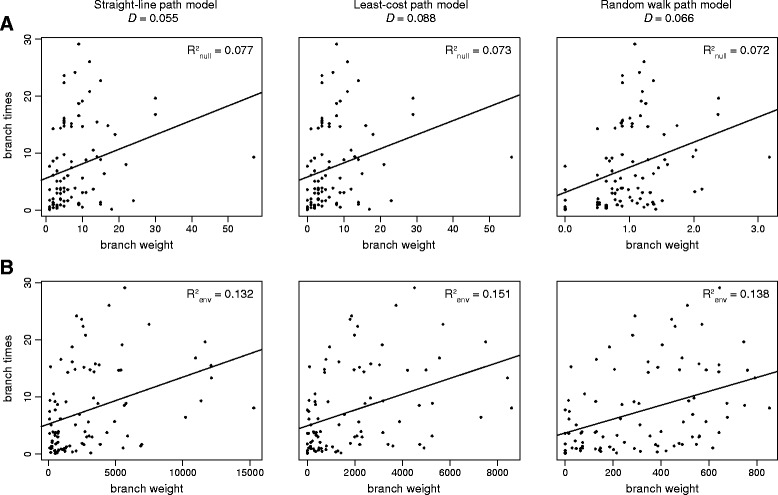
Linear regressions between branch durations and branch weights for one phylogenetic tree. Plots in (**a**) were generated by calculating the branch weights using the “null” raster, whereas plots in (**b**) were generated by calculating the branch weights using the “elevation” raster treated as a resistance factor. Plots are shown for each of the three path models (straight line, least-cost, and random walk). The *D* value obtained under each path model is shown at the top. The plots show that the R^2^ of the regression is approximately doubled when spatial heterogeneity in elevation is taken into account

Figure 4 displays the linear regressions between branch durations and branch weights for a rabies virus phylogeny randomly chosen from the posterior tree distribution. Regressions are shown for “weights” calculated using the null raster (Fig. 4a) and for “weights” calculated using the elevation raster, when elevation is treated as a factor that impedes movement (Fig. 4b). Results are shown for each of the three path models (straight-line, least-cost, and random walk models). In this example, there is comparatively little difference among path models in the strength of the relationship between branch durations and branch weights. For all three path models, R^2^_env_ is approximately twice as great as R^2^_null_, so the corresponding *D* values are positive (0.132 for the straight-line model; 0.151 for the least-cost model; 0.138 for the random walk model). Thus while geographic distance alone explains a small part of among-lineage variation in velocity (i.e. R^2^_null_), this variation is better explained when the geographic elevation of the path taken by each lineage is taken into account (i.e. R^2^_env_). Table 2 reports *D* values for other environmental variables when applied to the same single phylogeny as that used in Fig. 4.

**Table 2:** Results of the randomisation tests on six environmental variables

Environmental variable	Path model	Treated as a resistance or conductance factor	Single-tree analysis: *D* value (*p*-value)	100 trees analysis: % of trees with *p* < 0.05
Croplands	straight-line	not applicable	−0.013 (0.43)	7 %
	least-cost	conductance	−0.069 (0.67)	0 %
	least-cost	resistance	0.014 (0.39)	28 %
	random walk	conductance	−0.061 (0.35)	1 %
	random walk	resistance	0.012 (0.28)	27 %
Forests	straight-line path	not applicable	0.001 (0.42)	0 %
	least-cost path	conductance	−0.046 (0.84)	0 %
	least-cost path	resistance	0.013 (0.49)	2 %
	random walk path	conductance	−0.064 (0.72)	3 %
	random walk path	resistance	−0.035 (0.91)	0 %
Savannas	straight-line path	not applicable	−0.075 (0.89)	0 %
	least-cost path	conductance	0.018 (0.17)	1 %
	least-cost path	resistance	−0.059 (0.75)	0 %
	random walk path	conductance	−0.003 (0.81)	4 %
	random walk path	resistance	−0.072 (0.96)	0 %
Elevation	straight-line path	not applicable	0.055 (0.02*)	52 %
	least-cost path	conductance	−0.072 (0.86)	0 %
	least-cost path	resistance	0.078 (0.00*)	81 %
	random walk path	conductance	−0.061 (0.44)	0 %
	random walk path	resistance	0.067 (0.10)	72 %
Human density	straight-line path	not applicable	−0.076 (0.94)	0 %
	least-cost path	conductance	0.063 (0.04)	17 %
	least-cost path	resistance	−0.069 (0.90)	0 %
	random walk path	conductance	−0.072 (0.99)	12 %
	random walk path	resistance	−0.070 (0.78)	0 %
Inaccessibility	straight-line path	not applicable	0.070 (0.00*)	18 %
	least-cost path	conductance	−0.047 (0.84)	0 %
	least-cost path	resistance	0.089 (0.00*)	51 %
	random walk path	conductance	−0.060 (0.66)	0 %
	random walk path	resistance	0.077 (0.18)	35 %

For each combination of environmental variable and path model, the test is applied to a single phylogeny, and to a set of 100 trees. For the former, the *D* statistic and *p*-value of the test are shown. For the latter, we report the percentage of trees for which *p* < 0.05

(*) *p*-value < 0.05

**Table 3:** Investigating the performance of the null hypothesis randomisation test. Sets of artificial data sets were created using four different approaches

	Path model		
Approach for creating artificial data sets:	straight-line	least-cost	random walk
(i) branch durations randomly permutated among branches	6 %	3 %	3 %
(ii) branch durations equal to spatial distance	0 %	0 %	5 %
(iii) branch durations equal to environmental “weight”	100 %	100 %	100 %
(iv) branch durations equal to a value between (ii) and (iii)	95 %	100 %	100 %

In each case, artificial data sets were applied to the “elevation” raster treated as a resistance factor. Values equal the percentage of 100 artificial data sets for which the *p*-value of the null hypothesis test was < 0.05

The statistical significance of each *D* value can be assessed by comparing it to a null distribution generated by randomising the empirical node positions (see Step 5 in Methods). [Validation of this randomisation procedure on simulated data sets is provided in the next section.] In each case, 100 randomisation replicates were performed. Table 2 reports the *p*-values obtained when this randomisation procedure is applied to the rabies virus phylogeny introduced above. For this data set only some of the *D* values are statistically significant. Specifically, significant positive *D* values were obtained for the “elevation” and “inaccessibility” rasters (when treated as resistance factors) under the least-cost path model (Table 2).

Although these results are interesting they are based on only one tree and therefore do not take in account the statistical uncertainty arising from phylogenetic inference. We will now demonstrate how the null hypothesis test is performed on 100 phylogenies sampled from the posterior distribution of trees. First, we estimated *D* values for each of the 100 trees. This generates, for each combination of environmental variable and path model, a distribution of 100 *D* values. The randomisation procedure is then applied to each *D* value, precisely as outlined above. This results in 100 *p*-values for each raster/path model combination. We therefore report the percentage of trees which give rise to *p*-values < 0.05 (Table 2); this can be interpreted as the posterior probability of observing a significant correlation between lineage movements and the environmental variable.

**Fig. 5:**
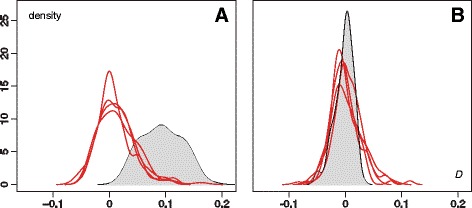
Empirical distributions of the *D* statistic (in grey), calculated from 100 trees sampled using Bayesian MCMC inference. These are compared with five replicates of the null distribution of *D* generated by the randomisation procedure (red lines). In (**a**) the distributions were calculated using the “elevation” raster (as a resistance factor) and in (**b**) they were calculated using the “forests” raster (as a conductance factor). In both cases the least-cost path model was used. For visual clarity, discrete histograms were converted into density curves using a Gaussian smoothing kernel

We illustrate this procedure in Fig. 5, which compares the empirical distribution of *D* (grey) with five replicates of the null distribution of *D* generated by the randomisation procedure (red lines). In Fig. 5a the distributions were calculated using the “elevation” raster (as a resistance factor) and in Fig. 5b they were calculated using the “forests” raster (as a conductance factor). In both cases the least-cost path model was used. In Fig. 5a, the empirical distribution is greater than the randomisation replicates, indicating that the environmental variable in question explains viral lineage movement, whereas in Fig. 5b there is no noticeable difference between the empirical and null *D* values.

The environmental variables in Table 2 can be grouped into three categories: (i) rasters that have little or no association with lineage movement (i.e. *p* < 0.05 for less than 5 % of trees), (ii) rasters for which there is weak evidence that they affect lineage movement (i.e. *p* < 0.05 for more than 5 % but less than 50 % of trees), and (iii) rasters for which there relatively stronger evidence that the environmental variable is associated with slower or faster movements (i.e. *p* < 0.05 for more than 50 % of trees). The “forests” and “savannas” rasters fall within the first category, whilst “croplands” (as a resistance factor) and “human population density” (as a conductance factor) belong to the second category. Only the “elevation” raster (as a resistance factor) clearly belongs to the third category. The importance of the “inaccessibility” raster (as a resistance factor) is less clear and is sensitive to the path model chosen. For both the “elevation” and “inaccessibility” rasters, the fraction of trees with *p* < 0.05 is smaller under the straight-line path model than under the other two path models. This may reflect the over-simplified nature of the straight-line model, which permits biologically unrealistic scenarios, such as paths that traverse large water bodies.

### Performance on simulated data sets

Table 3 shows the results of null hypothesis tests performed on artificial data sets. These data sets were simulated using three different approaches. Simulation approaches (i) and (ii) generate artificial data sets under the null hypothesis (such that branch duration is independent of the environmental variable in question). Therefore for these two approaches the fraction of significant results should equal the critical value of the test. This is indeed the case, as only 0–7 % of tests on artificial data produce a *p*-value < 0.05 and hence the type I error of the test appears to be appropriate. Simulation approaches (iii) and (iv) generates data sets under the alternate hypothesis, i.e. branch duration is equal or influenced by the environmental raster. The null hypothesis was almost always rejected (at *p* < 0.05) when the test was applied to data generated using approaches (iii) or (iv), indicating that the test has reasonable statistical power in the context of this data set. Note that very similar results were also obtained on data sets whose simulations were based on alternative environmental rasters (e.g. croplands, inaccessibility; results not shown).

## Conclusion

In this study we show that it is possible to integrate genomic and environmental data in order to test hypotheses concerning the mode and tempo of virus dispersal during emerging epidemics. The raccoon rabies data set explored here was chosen for illustrative purposes and our results strongly support the notion that increasing elevation is associated with slower movements of this virus. This result is biologically plausible and the impact of elevation on the dissemination of raccoon rabies was previously addressed by Biek et al. [25]. Their analysis used spatial kriging to compute annual contours from the date of the first reported case of each county and the contours were coloured according to temporal periods identified by phylogenetic molecular clock analysis. They then visually compared the resulting contours overlaid on an elevation map, enabling them to posit that mountain ranges likely formed a barrier to raccoon movement. We intend to explore the application of our new method to other viral outbreaks in future work.

We have implemented three path models in our analytical framework. Although the straight-line path model is perhaps overly simplistic, the appropriateness of one model over another will depend on the locomotive behaviour of the pathogen’s host species (and of its insect vector, if it has one). In the case of the raccoon rabies epidemic, we had no prior assumption about which path model might be most appropriate and therefore we decided to test them all. In other instances, there may be good reasons to choose *a priori* one path model over another. For instance, one might chose the least-cost path model over random walk path model in some cases, for example viruses carried by migratory birds; i.e. the least-cost model may be more appropriate when host-species intentionally reach specific locations by avoiding non-suitable landscape areas.

Simulations indicate that the randomisation procedure used to test the null hypothesis (i.e. no association between lineage movement and an environmental variable) has appropriate type I error rates and acceptable statistical power (Table 2). However, it is important to note that a significant result represents evidence only for a *correlation* between virus dispersal and an aspect of the geographic landscape. In future work we intend to explore whether generalized linear models can be applied to this framework in order to account for possible correlations among the environmental variables in question. In the meantime, the broader ecology and epidemiology of the pathogen, host, and habitat concerned should be taken into account when interpreting results.

Currently, our analytical framework does not allow for the analysis of environmental variables, such as temperature or humidity, that vary notably over the course of the epidemic under investigation. The assumption of constant values is unlikely to be unrealistic for the environmental variables investigated here, although some variation has undoubtedly occurred in human population density. The extension of our approach to time-varying environments is theoretically feasible, but poses significant technical and practical problems, specifically (i) the acquisition of a series of environmental rasters that represents change in the variable concerned at a sufficiently precise temporal resolution, and (ii) generalisation of the least-cost and random-walk path models to three rather than two dimensions, with the third dimension corresponding to rasters that represent different points in time. A second improvement for future work would be to allow the co-analysis of several environmental factors in a multivariate framework. Such a framework would require correlation statistics, for example those based on a generalised linear model (GLM), as in [22].

### Implementation

The methods introduced here are implemented in R (R Core Team 2015 [32]) and the scripts are freely available from http://evolve.zoo.ox.ac.uk/Evolve/Software.html. Example files and a tutorial are available as Additional file 1.

## Additional file

Additional file 1:
** Example files and tutorial related to the present study.** (ZIP 36167 kb)
