# Supplementary material for: Explaining the geographic spread of emerging epidemics: a framework for comparing viral phylogenies and environmental landscape data
Source: BMC Bioinformatics. 2016 Feb 11;17:82. doi: 10.1186/s12859-016-0924-x (PMC4750353; doi:10.1186/s12859-016-0924-x)
Supplement: Additional file 1: — Example files and tutorial related to the present study. (ZIP 36167 kb) [file 12859_2016_924_MOESM1_ESM.zip › Appendix/Tutorial_RABV_raccoon.pdf]

Appendix of Dellicour *et al.*: a tutorial for using the R scripts in order to study the rabies spread in North American raccoon populations

Simon Dellicour

January 12, 2016

The present tutorial describes how to use the R scripts to study the impact of a particular environmental layer (an "elevation" raster) on the dispersal velocity of the rabies (RABV) epidemic in North American raccoon populations [1]. The first step is to download the package `seraphim` (`http://evolve.zoo.ox.ac.uk/Evolve/Software.html`) and place the "seraphim_1.0.beta.tar.gz" file in a R workspace directory. The package can then be installed from this archive file using the following R command:

```
> install.packages("seraphim_1.0.beta.tar.gz", repos=NULL, type="source")
```

Once installed, the package has to be loaded. Note that to be loaded, this package requires the preliminary installation of the following R packages: "ape", "doMC", "fields", "gdistance", "gstat", "ks", "MASS", "raster", "rgdal"[1] and "vegan". To load the `seraphim` package, simply enter:

```
> library(seraphim)
```

This tutorial requires the following example files: "BEAST_RABV_raccoons.trees", a file containing the phylogenetic trees inferred for the RABV dataset using the method of Lemey *et al.* [2], and "Elevation_RABV_raccoons.asc", the environmental layer "elevation".

[1] The "rgdal" package requires the preliminary installation of GDAL (Geospatial Data Abstraction Library), a C++ library for reading and writing raster geospatial data formats. See `http://www.gdal.org` for further details.

Step 1: extracting spatio-temporal information in trees

The first step is to extract the spatio-temporal information contained in the phylogenetic trees previously inferred by the continuous phylogeographic method implemented in BEAST [2] (the BEAST ”xml” input file is also provided with this tutorial). The tree file “BEAST_RABV_raccoons.trees” contains 11001 trees sampled by the MCMC chain. We will here use the “treeExtractions” function of the package `seraphim` to extract the information contained in 100 post burin-in trees sampled in this posterior distribution. The “treeExtractions” function first requires the definition of the following parameters: “localTreesDirectory” (name of the directory to create and where spatio-temporal information contained in each tree will be saved), “allTrees” (name of the “.trees” file), “burnIn” (number of trees to discard as burn-in, i.e. a number defining a series of first trees in which no tree will be sampled), “randomSampling” (boolean variable specifying if the trees have to be randomly sampled or sampled at the largest possible regular interval), “nberOfTreesToSample” (number of trees to sample), “mostRecentSamplingDatum” (most recent sampling datum in a decimal format) and “coordinateAttributeName” (attribute name used to indicate the geographic coordinates within the trees file).

```
> localTreesDirectory = "Extracted_trees"
> allTrees = scan(file="BEAST_RABV_raccoons.trees", what="", sep="\n",
quiet=TRUE)
> burnIn = 1001
> randomSampling = FALSE
> nberOfTreesToSample = 100
> mostRecentSamplingDatum = 2004.7
> coordinateAttributeName = "location"
```

Once all these parameters have been specifying, the “treeExtractions” function can be launched as follows:

```
> treeExtractions(localTreesDirectory, allTrees, burnIn, randomSampling,
nberOfTreesToSample, mostRecentSamplingDatum, coordinateAttributeName)
```

Step 2: estimating dispersion/epidemiological statistics

The second step of this tutorial consists in using the extracted information to estimate a series of epidemiological statistics using the “spreadStatistics” function. So far, estimations of three statistics are available: the mean dispersal velocity, the mean diffusion coefficient as defined by Pybus *et al.* [3], and the evolution of the maximal wavefront distance. The function will both estimate values and generate/save graphs. It simply requires the user to specify (i) the directory in which extracted spatio-temporal information has been saved (see above), (ii) the number of extraction of files to use (this number cannot be higher that the number of extractions performed in the previous step), (iii) the “onlyTipBranches” boolean variable indicating if statistics estimations have to be

based on the tip branches only[2], (iv) the “showingPlots” boolean variable specifying if the function has to display the different graphs (in both cases, all the graphs will be saved as pdf files), and (v) the “outputName” string that will be used as a prefix to name the different outputs of the function.

```
> nberOfExtractionFiles = 100
> spreadStatistics(localTreesDirectory, nberOfExtractionFiles,
onlyTipBranches=FALSE, showingPlots=TRUE,
 outputName="RABV_raccoons")

Median value of average dispersal velocity = 37.00252
95% credible region = [21.32282, 164.84]
Median value of diffusion rate = 1126.53
95% credible region = [641.8642, 7975.519]
```

As displayed in Figure 1, the function will also generate and save four different graphs: the kernel density estimates of dispersal velocity parameters (dispersal velocity variation among lineages vs. $\log_{10}$[mean dispersal velocity]), the kernel density estimates of diffusion coefficient parameters (diffusion coefficient variation among lineages vs. $\log_{10}$[mean diffusion coefficient]), as well as the evolution of the spatial and patristic maximal wavefront distances from epidemic origin. The spatial maximal wavefront distance corresponds to the straight-line distance (i.e. “as the crow flies”) from the estimated location of the root, and the patristic maximal wavefront distance corresponds to a distance computed as the sum of geographical distances associated with each branch connecting a given node to the root.

Step 3: preliminary analysis of environmental layers

When we have several different environmental rasters to test, this is useful to preliminary investigate which ones are potential resistance or conductance factors and then focus on a restricted set of selected raster files. This first analysis is directly based on the environmental raster cell values and without performing any randomisation steps. When the number of randomisation steps is set to zero, the function simply estimates the correlation between dispersal durations and environmental weights associated with each phylogenetic branch. In the context of this tutorial, we will estimate the correlation between the dispersal durations and the environmental weights computed for each branch using the least-cost method [4, 5] and while treating the “elevation” raster as a potential resistance factor. Note that when we do not have any prior information about the impact of environmental variable, it might make sense to test each factor once as a resistance and once as a conductance variable. The different parameters of the “spreadFactors” function have to be specified as follows:

```
> envVariables = list(raster("Elevation_RABV_raccoons.asc"))
> resistances = c(TRUE)
```

[2]This option could be useful for example to investigate the potential impact of phylogenetic uncertainty on the statistic values.

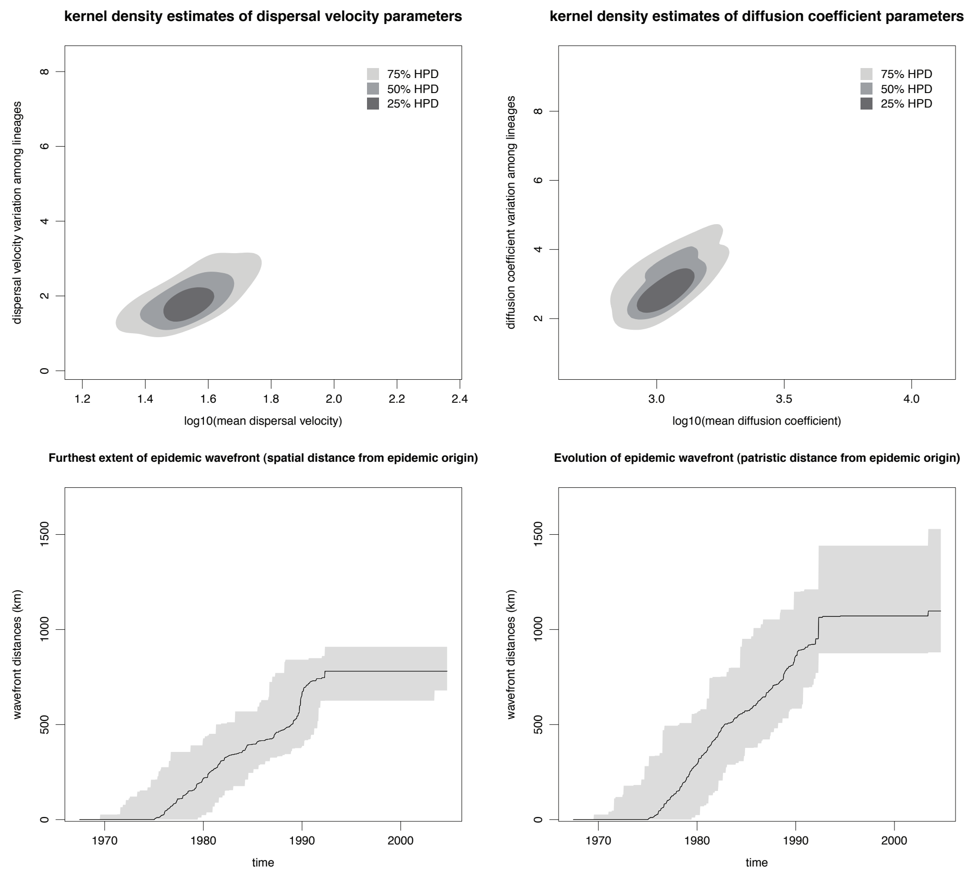

Figure 1: estimated epidemiological statistics. For the two first graphs, the three contours show, in shades of decreasing darkness, the 25%, 50%, and 75% HPD regions via kernel density estimation. For the two last graphs, grey area corresponds to 95% credible regions of the estimated wavefront position.

```
> pathModel = 2
> nberOfRandomisations = 0
> randomProcedure = 1
> variogramModels = list()
> outputName = "Elevation_least-cost"
> showingPlots = FALSE
> nberOfCores = 1
> OS = "Unix"
```

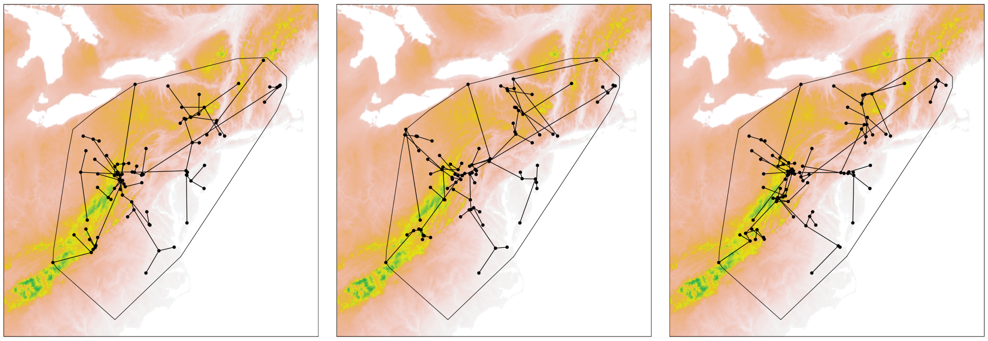

Figure 2: the three first sampled trees mapped on the elevation raster. The contours correspond to the convex hull defined by all the sampled trees.

Even if in this particular case we focus on only one raster file, the "envVariables" object has to be a list of raster files and the "resistances" object has to be a vector of boolean variables specifying if each raster has to be treated as a resistance ("TRUE") or a conductance ("FALSE") variable. The "pathModel" variable specifies which path taken model has to be used to compute the environmental "weights" associated with each branch: "1" (straight-line path model), "2" (least-cost path model [4, 5]) or "3" (random walk model [6]). The "randomProcedure" value and "variogramModels" list are not important at the moment but have to be created; simply set them equal to one and to an empty list, respectively (the variogram model for each tested raster is necessary when using the raster simulations procedure and can be estimated using the "variogramModel" function of this package; see the package manual for further details). As for the "spreadStatistics" function, the "outputName" string will be used as a prefix to name the different outputs of the function. If the boolean parameter "showingPlots" equals "TRUE", the function will generate and save several graphs like the one displayed in Figure 2 (but in that case, the function will run much slower). Finally, we also have to specify the number of cores ("nberOfCores") to use and the operating system on which the function will run. At this stage, parallelisation of the code is not useful and then simply set the number of cores to one. The information about the nature of the operating system is only useful when the function has to call the "Circuitscape" Python package [6] (see the `seraphim` manual for further details). Before launching the function, we will modify the raster cell values so that minimum raster cell values equal to one instead of zero (note that this operation will not affect cells with a "no data" value). For that purpose, we will add a value of one to all the cells (except the ones with a "no data" value):

```
> envVariables[[1]][] = envVariables[[1]][] + 1
```

The aim of this modification is to allow a comparison with an artificial raster with all the cell values equal to one. This "null" raster will be used to compute spatial distances with the selected path taken model (straight-line, least-cost or random walk path model). In addition to environmental weight(s), each phylogenetic branch will then be also associated with a geographical distance measured in a number of raster cells. After this step, the function can be launched using the following command:

```
> spreadFactors(localTreesDirectory, nberOfExtractionFiles, envVariables,
```

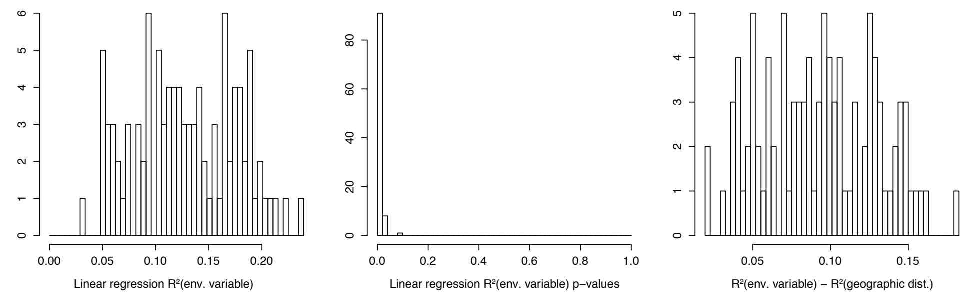

Figure 3: results of linear regressions performed between dispersal durations and resistance values computed on the elevation raster. Each value corresponds to one sampled tree.

```
resistances, pathModel,  nberOfRandomisations, randomProcedure,
outputName, variogramModels, showingPlots, nberOfCores, OS)
```

The function will generate two outputs: (i) a text file containing some statistic values (one per statistic and per sampled/extracted tree) measuring the correlation between dispersal durations and environmental weights computed for each branch, (ii) as well as a pdf file presenting histograms of the distributions of these different statistics (Fig. 3). These statistics are for instance the "environmental" determination coefficient (estimated from the linear regression between the dispersal durations and the environmental weights associated with each branch), the environmental determination coefficient p-value, and the difference D (difference between the environmental determination coefficient and the "spatial" determination coefficient estimated from the linear regression between the dispersal durations and the geographical distances associated with each branch). Note that, as mentioned above, the geographical distance is computed using the selected path taken model on a "null" raster with uniform cell values equal to one. As we can see in Figure 3, the distribution of determination coefficients differences clearly tends to be different from zero.

Step 4: tests based on a randomisation procedure

The final step is to test the level of significance of the statistics estimating the correlation between dispersal durations and environmental weights computed for each branch and based on the "elevation" raster. In this example, we will use the randomisation of phylogenetic node positions, which was already specified above ("randomProcedure = 3"). First, we have to create a new "envVariables" list containing the new resistance raster and specify the number of randomisation steps we want to perform:

```
> nberOfRandomisations = 100
```

Once these new parameters are specified, the "spreadFactors" function can be re-launched:

```
> spreadFactors(localTreesDirectory, nberOfExtractionFiles, envVariables,
resistances, pathModel,  nberOfRandomisations, randomProcedure,
outputName, variogramModels, showingPlots, nberOfCores, OS)
```

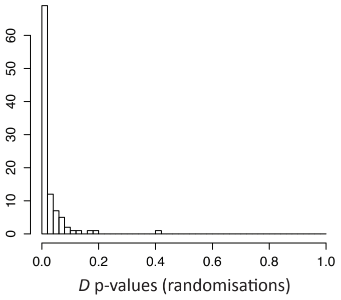

Figure 4: results of the randomisation test performed to test the siginificativity of the D statistic. Each p-value corresponds to one sampled tree.

The randomisations step can be very time consuming (especially when analysing several rasters and/or when selecting the random walk model). In that case, "the spreadFactors" function can run on several cores by specifying a number of cores higher than one with the "nberOfcores" parameter. The parallelisation is performed using the "doMC" package and only works with an R script launched from a terminal (on Unix terminal, use the command "R < myScript.r –no-save"). Again, the function will generate and save a text file and a pdf file. Figure 4 displays the histograms of p-values (one per sampled tree) obtained for the D statistic. As we can see on the graph, the majority of p-values are higher than 0.05. To know the exact number, e.g., the number of p-values smaller than 0.05 estimated from the comparison between randomised and observed D values:

```
> tab = read.table("Elevation_least-cost_randomisation_results.txt",
header=T)
> a = tab[,"Uni_LR_delta_R2_p.values_Elevation_RABV_raccoons_R"]
> print(length(a[a[]<0.05]))
```

```
81
```

Based on the randomisation of phylogenetic node positions, 81 out of 100 sampled trees are associated with a significant p-value. This number of p-values smaller than 0.05 mean that for 81% of the trees sampled in the posterior distribution, elevation is significantly associated with a slower rabies spread in North American raccoon populations.

References

[1] Biek R, Henderson JC, Waller LA, Rupprecht CE, Real LA (2007). A high-resolution genetic signature of demographic and spatial expansion in epizootic rabies virus. *PNAS* 104: 7993-7998.

[2] Lemey P, Rambaut A, Welch JJ, Suchard MA (2010). Phylogeography takes a relaxed random walk in continuous space and time. *Molecular Biology & Evolution* 27: 1877-1885.

[3] Pybus OG, Suchard MA, Lemey P, Bernardin FJ, Rambaut A, Crawford FW, Gray RR, Arinaminpathy N, Stramer SL, Busch MP, et al. (2012). Unifying the spatial epidemiology and molecular evolution of emerging epidemics. *Proceedings of the National Academy of Sciences of the United States of America* 109(37):15066-15071.

[4] Dijkstra EW (1959). A note on two problems in connexion with graphs. *Numerische Mathematik* 1: 269-271.

[5] Van Etten J (2012). R package gdistance: distances and routes on geographical grids. R package version 1.12.

[6] McRae BH (2006). Isolation by resistance. *Evolution* 60: 1551-1561.
